# Age- and Sex-Specific Reference Intervals for TSH, FT4, and FT3 Derived from the Turkish Multi-Center Cohort

**DOI:** 10.3390/diagnostics16121800

**Published:** 2026-06-11

**Authors:** Gülce Kiren, Gizem Yılmaz Çalık, Merve Güngören, Hikmet Can Çubukçu

**Affiliations:** 1 Department of Medical Biochemistry, Ministry of Health Sincan Training and Research Hospital, Ankara 06949, Türkiye; hikmetcancubukcu@gmail.com; 2 Department of Medical Biochemistry, Ministry of Health Pursaklar State Hospital, Ankara 06145, Türkiye; gizemyilmazcalik@gmail.com; 3 Department of Medical Biochemistry, Ministry of Health Mamak State Hospital, Ankara 06270, Türkiye; merve.durak@gmail.com

**Keywords:** reference interval, indirect method, refineR, thyroid-stimulating hormone, free thyroxine, free triiodothyronine

## Abstract

**Background/Objectives:** Reference intervals (RIs) for thyroid-stimulating hormone (TSH), free thyroxine (FT4), and free triiodothyronine (FT3) are essential for accurate interpretation of thyroid function tests. Given the pronounced age- and sex-related biological variability of these analytes, locally derived and population-specific RIs are required. **Methods**: This multicenter study estimated age- and sex-specific RIs for TSH, FT4, and FT3 using an indirect statistical approach applied to routine laboratory information system data from three hospitals in Ankara, Türkiye. Measurements were performed on Maglumi X8 and X6 analyzers (Snibe Diagnostic, Shenzhen, China) using chemiluminescent immunoassays. Reference intervals were calculated using the refineR algorithm implemented in the R programming language, with Box–Cox or shifted two-parameter (mod) Box–Cox transformations selected according to data distribution. **Results**: More than 100,000 test results were included after predefined exclusions. In adults (≥18 years), the estimated TSH reference intervals were 0.495–5.83 mIU/L in males and 0.485–6.40 mIU/L in females, with higher upper limits observed in females. Adult FT4 reference intervals were approximately 12.9–21.5 pmol/L, and FT3 intervals were 3.46–6.04 pmol/L in males and 3.46–5.76 pmol/L in females. Pediatric reference intervals for TSH and FT4 were also derived across predefined age categories, demonstrating age-dependent variation. **Conclusions**: This study establishes age- and sex-stratified reference intervals for TSH, FT4, and FT3 using large multicenter routine laboratory datasets, encompassing both adult and pediatric populations. The findings highlight limitations of manufacturer-provided intervals and support the application of indirect statistical approaches, such as refineR, for local reference interval determination in clinical laboratories.

## 1. Introduction

Reference intervals (RIs) are used by clinical laboratories and physicians to distinguish physiological from pathological test results [1,2]. Therefore, establishing appropriate RIs for each analyte is essential for both clinical laboratories and assay manufacturers, as they provide reliable benchmarks for patient care, reduce unnecessary investigations, and support the overall quality of laboratory medicine. The Clinical and Laboratory Standards Institute (CLSI) provides recommendations for defining RIs. In the so-called “direct” approach, an RI is determined using samples collected from a predefined reference population (at least 120 individuals) according to specified inclusion criteria [3]. These recommendations reflect the need for RIs used in clinical decision-making to account for population characteristics (age, sex, ethnicity, genetic and environmental factors) as well as analytical variation across measurement systems [4,5]. However, the direct method is often impractical in routine settings because identifying an appropriate reference population is challenging and the process is costly and time-consuming [6,7]. Indirect methods offer an alternative by leveraging routine laboratory information system data and statistical modeling to estimate RIs from mixed clinical datasets [4]. The refineR algorithm, recently introduced by Ammer et al. [8], is an indirect approach that models the non-pathological component using Box–Cox-based transformations with bootstrap-derived uncertainty estimates. In the present study, we applied refineR (R programming language) to thyroid function tests, thyroid-stimulating hormone (TSH), free thyroxine (FT4), and free triiodothyronine (FT3), which are among the most frequently ordered assays and exhibit substantial biological variability by age, sex, and analytical platform [9]. Accordingly, population-specific, partitioned RIs are required for reliable interpretation. We therefore derived age- and sex-stratified RIs for these analytes using large routine datasets generated on MAGLUMI X6 and X8 analyzers (Snibe Diagnostic, Shenzhen, China) in multiple hospitals in Ankara, Türkiye, and compared the estimated RIs with the manufacturer’s intervals.

## 2. Materials and Methods

### 2.1. Study Design

**Figure 1:**
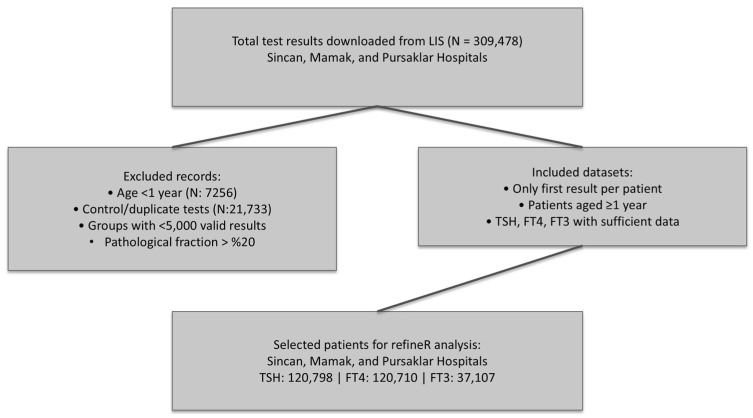
Flowchart of the study design showing data extraction from laboratory information systems, applied exclusion criteria, and the final selection of datasets used for refineR-based reference interval estimation for TSH, free T4, and free T3. LIS, laboratory information system; TSH, thyroid-stimulating hormone; FT4, free thyroxine; FT3, free triiodothyronine; RI, reference interval; LRL, lower reference limit; URL, upper reference limit; CI, confidence interval; y, years.

This study used an indirect approach to estimate RIs from routine laboratory data obtained from three hospitals in Ankara, Türkiye. In accordance with recommendations by Ammer et al. [8], only the first result per patient was included; repeated measurements, patients younger than 1 year, and partitions with fewer than 5000 results were excluded. The study design and data selection workflow are summarized in Figure 1.

### 2.2. Subjects

The study was approved by the ethics committee of the Sincan Training and Research Hospital (SEAH-BAEK2025-54). Data extraction and analysis were performed after removing all personal identifiers.

Laboratory information system (LIS) data from Sincan Training and Research Hospital, Mamak State Hospital, and Pursaklar State Hospital (Ankara, Türkiye) were retrieved for analysis.

Records from patients younger than 1 year were excluded. Age- and sex-based partitions were predefined as follows: for TSH, pediatric (1–<14 years and 14–<18 years) and adult (≥18 years) groups stratified by sex; for FT4, adult groups stratified by sex, pediatric groups aged 1–<15 years stratified by sex, and an adolescent group aged 15–<18 years analyzed as a combined-sex cohort; and for FT3, adult (≥18 years) groups stratified by sex. We aimed to include at least 5000 results per partition; partitions below this threshold were not modeled because RI estimation was considered unreliable.

### 2.3. TSH, FT4, and FT3 Measurement Procedures

Analyses were performed using three Snibe MAGLUMI X8 analyzers at Sincan Training and Research Hospital, two Snibe MAGLUMI X6 analyzers at Pursaklar State Hospital, and three Snibe MAGLUMI X6 analyzers at Mamak State Hospital (Snibe Diagnostics, Shenzhen, China). Measurements were obtained with chemiluminescent immunoassays, including a second-generation TSH assay, a first-generation free T4 assay, and a second-generation free T3 assay. In all laboratories, blood samples were collected in gel-containing serum separator tubes, allowed to clot, and centrifuged; separated serum was used for analysis. Limits of detection were 0.006 mIU/L for TSH, 1.93 pmol/L for FT4, and 0.61 pmol/L for FT3.

TSH traceability was established against the WHO Third International Standard (81/565). FT4 traceability was standardized against United States Pharmacopeia (USP) standards, and FT3 traceability against the USP reference standard (catalog no. 1368008). Coefficients of variation (CV, %) at two quality control levels are provided in the Supplementary Materials.

Analytical performance was ensured through daily two-level internal quality control and regular participation in the RIQAS external quality assessment (EQA) program.

### 2.4. Reference Interval Estimation

During RI estimation using refineR, the proportion of pathological results was targeted to remain below 20% based on International Classification of Diseases-10th Revision (ICD-10)-coded thyroid diseases. All ICD-10 E00–E07 subcategories corresponding to disorders of the thyroid gland were considered for this estimate, and the complete code list is provided in the Supplementary Materials. Adult TSH, FT3, and FT4 results were analyzed separately for females and males. For pediatric TSH and FT4, we adopted age partitions and combined-sex grouping similar to those used by Adeli et al. [10] in a direct-method study on MAGLUMI X8 analyzers (Snibe Diagnostic, Shenzhen, China). The FT4 adolescent group aged 15–18 years was also analyzed as a combined-sex cohort because sex-specific subgroup sizes did not meet the predefined minimum sample size threshold for reliable RI estimation.

Reference intervals for each subgroup were estimated using the refineR package in R version 4.4.2. For each fitted model, 200 bootstrap iterations were used to estimate the lower (2.5th percentile) and upper (97.5th percentile) reference limits and their 95% confidence intervals. The distance ratio was calculated for each subgroup. For skewed distributions (distance ratio 0.25–0.75) and heavily skewed distributions (distance ratio <0.25), the modified Box–Cox model (modBox–Cox) was applied. Estimated RIs were compared with the manufacturer-provided intervals.

## 3. Results

The number of patient records obtained from each hospital is presented in Figure 1. The following features were obtained from the aforementioned three hospitals: analyte name, measurement unit, numerical result, analysis date, patient age, sex, and a unique anonymized patient identifier.

Data were collected from January 2025 to July 2025. As shown in Table 1, all partitions met the recommended sample size (>5000), except for pediatric FT3, and the pathological fraction based on ICD-10-coded thyroid diseases remained below the suggested 20% threshold, staying under 10% in our dataset. Reference intervals were estimated using refineR with the predefined age- and sex-based partitions: TSH in pediatric (1–<14 and 14–<18 years) and adult (≥18 years) groups stratified by sex; FT4 in adult groups stratified by sex, in females and males aged 1–<15 years, and in a combined-sex cohort aged 15–<18 years; and FT3 in adults (≥18 years) stratified by sex (Table 1).

**Figure 2:**
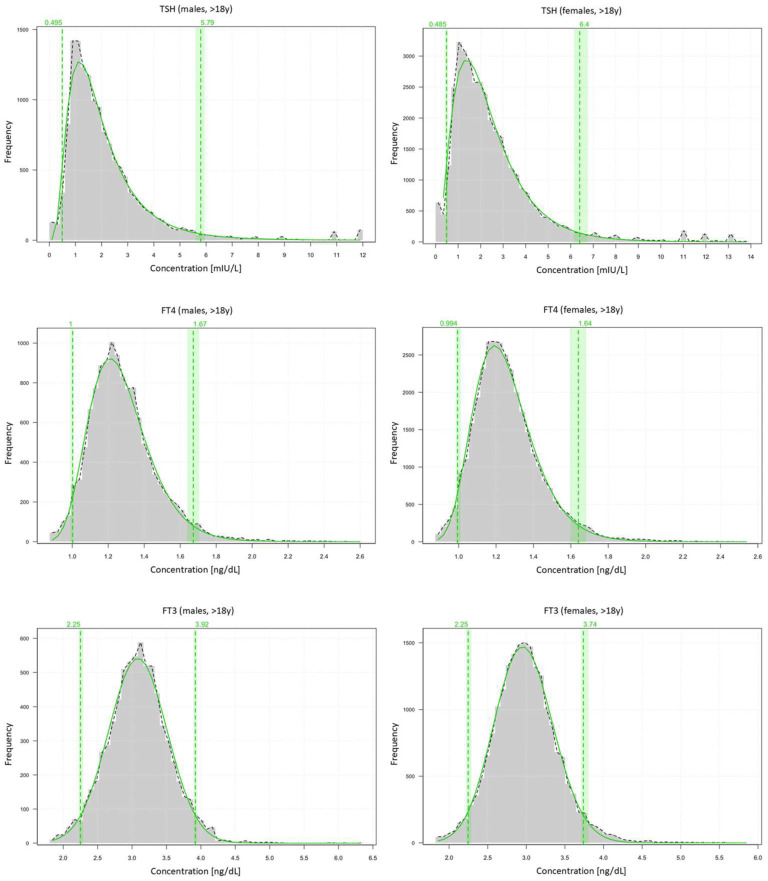
Graphical output of the refineR algorithm for TSH, free T4, and free T3 in adult female and male groups. illustrating the observed data distribution (histograms) and the estimated reference distribution for non-pathological results (solid line). Lower and upper reference limits are indicated by vertical dashed lines, with shaded regions showing the 95% confidence intervals.

The graphical representations of the estimated models for TSH, free T4, and free T3 in adult female and male groups are presented in Figure 2. Graphical outputs for TSH and free T4 across the predefined pediatric age categories are provided in Supplementary Figure S1.

Among the TSH subgroups, the standard one-parameter Box–Cox transformation was adequate for adult males (≥18 years; distance ratio 0.799). In contrast, the distributions for adult females (≥18 years), children (1–<14 years), and adolescents (14–<18 years) were skewed; therefore, the two-parameter modified Box–Cox model (refineR option: model = “modBox–Cox”) was applied (Table 2).

**Table 2:** Overview of estimated RIs for TSH, Free T3, and Free T4 tests.

Test	Group	*n*	Distance Ratio	Model	LRL (95% CI)	URL (95% CI)	Manufacturer İnterval
TSH (mIU/L)	Children (1–14 y)	9662	0.000 (heavily skewed)	modBoxCox	0.387 (0.36; 0.73)	5.6 (5.49; 8.46)	
TSH (mIU/L)	Adolescents (14–18 y)	7399	0.000 (heavily skewed)	modBoxCox	0.634 (0.552–0.657)	6.19 (5.64–6.45)	
TSH (mIU/L)	Adult males (≥18 y)	27,111	0.799	BoxCox	0.495 (0.450–0.513)	5.83 (5.60–5.95)	0.3–4.5
TSH (mIU/L)	Adult females (≥18 y)	67,764	0.433 (skewed)	modBoxCox	0.485 (0.439–0.555)	6.40 (6.15–6.76)	0.3–4.5
FT3 (pmol/L)	Adult females (≥18 y)	25,638	0.000 (heavily skewed)	modBoxCox	3.46 (3.41–3.52)	5.76 (5.70–5.84)	3.07–6.45
FT3 (pmol/L)	Adult males (≥18 y)	9493	0.000 (heavily skewed)	modBoxCox	3.46 (3.43–3.52)	6.04 (5.99–6.07)	3.07–6.45
FT4 (pmol/L)	Female (1–<15 y)	11,776	0.737 (skewed)	modBoxCox	12.29 (10.67–13.13)	20.72 (20.21–21.24)	
FT4 (pmol/L)	Male (1–<15 y)	10,915	0.723 (skewed)	modBoxCox	11.73 (10.82; 13.13)	20.85 (20.21; 21.11)	
FT4 (pmol/L)	Adolescents (15–<18 y)	4760	0.000 (heavily skewed)	modBoxCox	12.75 (11.83–13.26)	21.36 (19.69–22.52)	
FT4 (pmol/L)	Female (≥18 y)	65,941	0.000 (heavily skewed)	modBoxCox	12.87 (12.67–13.00)	21.36 (20.59–21.62)	11.45–22.14
FT4 (pmol/L)	Male (≥18 y)	25,989	0.000 (heavily skewed)	modBoxCox	12.82 (12.74–13.00)	21.49 (21.11–22.01)	11.45–22.1

TSH, thyroid-stimulating hormone; FT3, free triiodothyronine; FT4, free thyroxine; LRL, lower reference limit; URL, upper reference limit; CI, confidence interval.

FT3 in adult females and males (≥18 years), as well as FT4 across all predefined partitions, required the shifted two-parameter Box–Cox transformation in refineR because the one-parameter form did not adequately correct the persistent right-skewness and inflation of upper percentiles. Estimated RIs, their confidence intervals, and the manufacturer’s intervals are presented in Table 2.

## 4. Discussion

To the best of our knowledge, this is the first study to establish pediatric and adult RIs for TSH and FT4, and adult RIs for FT3, on Snibe Maglumi analyzers using a multicenter dataset and an indirect statistical approach with the refineR algorithm.

Reported RIs for TSH in the literature show considerable variability, with lower limits generally ranging from 0.36 to 0.75 mIU/L and upper limits between 2.86 and 5.79 mIU/L. Specifically, studies using the indirect method reported RIs of 0.36–5.33 mIU/L [11], 0.21–2.86 mIU/L [12], and 0.58–5.79 mIU/L [13], while the study applying the direct method found an interval of 0.75–5.32 mIU/L [14].

In the present multicenter study, the estimated adult TSH RIs were 0.485–6.40 mIU/L in females and 0.495–5.83 mIU/L in males. These values differ from those reported by Lapić et al. [15], who established TSH RIs on a single Snibe MAGLUMI X6 analyzer using a direct method (0.77–5.04 mIU/L). The differences may reflect the use of an indirect method applied to a larger and more heterogeneous multicenter dataset in our study, which included four X6 and three X8 analyzers. Analytical and biological sources of variation (e.g., between-analyzer and within-analyzer variability, reagent lots, diurnal variation, and population characteristics) should be considered when interpreting these differences. Despite these factors, our estimates are broadly consistent with those reported by Ammer et al. [13], who also applied refineR to routine data and reported a TSH RI of 0.58–5.79 mIU/L. The agreement likely reflects the shared modeling strategy, in which Box–Cox transformations and bootstrap-based uncertainty estimation enable robust characterization of the non-pathological component in large mixed datasets, supporting the applicability of refineR to real-world laboratory distributions.

In our study, the upper reference limit for adult females was higher than that for adult males. This sex-related difference in TSH RIs has been reported previously. Estrogen may modulate TSH concentrations, and estrogen deficiency (e.g., in postmenopausal women) has been associated with higher TSH levels [16]. In addition, thyroid peroxidase antibody positivity is more frequent in women and may contribute to higher TSH concentrations [17]. Consistent with these observations, a meta-analysis by Xing et al. reported that mean TSH concentrations are approximately 0.27 mIU/L higher in women than in men [16]. Accordingly, our findings align with prior reports showing higher upper reference limits in females [11,18,19].

For FT4, the estimated RIs (approximately 12.87–21.49 pmol/L across partitions) were similar to those reported in adult cohorts from previous large indirect studies [13]. These values remained within the manufacturer’s suggested limits (11.45–22.14 pmol/L), supporting their analytical robustness for clinical interpretation.

For FT3, both adult male and female groups showed heavily skewed distributions (distance ratio 0.000), requiring the two-parameter modified Box–Cox model. The resulting intervals (3.46–6.04 pmol/L in males and 3.46–5.76 pmol/L in females) were narrower than, and fully contained within, the manufacturer’s broader range (3.07–6.45 pmol/L). These estimates were also consistent with reference intervals reported by Ammer et al. [13] using indirect methods (3.07–5.99 pmol/L).

In the pediatric population, the TSH RIs derived in this study (0.387–5.6 mIU/L for ages 1–<14 years and 0.634–6.19 mIU/L for ages 14–<18 years) were higher at both ends than those reported by Adeli et al. [10] in the Canadian CALIPER study (0.68–4.70 mIU/L and 0.44–3.73 mIU/L, respectively). Several factors may explain these differences. The CALIPER study used rigorously screened healthy volunteers to establish community-based pediatric RIs, whereas our analysis applied an indirect approach (refineR) to large sets of routine laboratory results rather than preselected healthy participants. Differences in population characteristics, inclusion criteria, and underlying health conditions may therefore have influenced the observed distributions [20]. In addition, regional and demographic factors such as iodine nutrition [21,22], genetic background [23,24], and population structure [25] can affect thyroid hormone levels. These methodological and biological differences likely contribute to the higher limits observed in the present study.

Dirks et al. [26] conducted a multicenter study across four immunoassay platforms (Abbott, Roche, Siemens, and Beckman Coulter) and reported that TSH RIs derived using indirect methods were generally consistent with those obtained using the direct method; however, notable between-analyzer differences were observed, particularly at the upper reference limits. The upper TSH limit was lowest with Abbott (4.06 mIU/L) and higher with Roche, Beckman, and Siemens analyzers (4.73, 4.71, and 4.84 mIU/L, respectively). The same study reported that FT4 RIs estimated by indirect methods were narrower than the manufacturer-provided intervals for the Roche and Siemens Atellica platforms. The indirectly derived FT4 ranges (Roche: 11.5–20.6 pmol/L; Siemens Atellica: 12.0–20.7 pmol/L) are largely consistent with our adult FT4 results. Collectively, these findings support the recommendation that manufacturer package-insert intervals should not be adopted uncritically and that laboratories should verify or establish intervals appropriate for their local populations.

Accordingly, the age- and sex-specific TSH RIs estimated in our study using an extensive Snibe MAGLUMI dataset were wider than the manufacturer-recommended range (0.3–4.5 mIU/L), consistent with the platform-related differences noted above and supporting the need for device- and population-specific RI derivation.

The main strengths of this study include the large, representative dataset and the low proportion of pathological cases (<10%) across all subgroups. The multicenter design enhances generalizability. Use of an indirect approach (refineR) enabled objective outlier handling and robust RI estimation, while age- and sex-stratified analyses captured relevant biological variation. Methodological consistency was maintained by using a single analyzer platform (Snibe MAGLUMI) with stable reagent lots across centers. Use of real-world clinical data increases practical relevance, and comparisons with published studies support the validity of our estimates. Overall, the indirectly derived RIs for TSH, FT3, and FT4 appear more representative for our local population than manufacturer-provided intervals.

In summary, using a large multicenter LIS dataset and an established indirect modeling approach (refineR), we derived age- and sex-partitioned RIs for TSH and FT4 and adult RIs for FT3 on the Snibe MAGLUMI platform. Differences between our estimated RIs and the manufacturer-provided intervals likely reflect a combination of population characteristics and assay- or platform-specific factors, underscoring the importance of local RI verification and, where feasible, local RI derivation. Strengths include the large sample size across most partitions, the multicenter design within a single analytical platform, and bootstrap-derived confidence intervals to quantify uncertainty. Limitations include restriction to a single geographic region, inability to reliably identify pregnancy status and use of drugs interfering with thyroid function tests, the cross-sectional design (precluding assessment of seasonal or long-term shifts), and inability to estimate pediatric FT3 RIs because of insufficient sample size, and the lack of formal sex-specific partitioning in some pediatric groups, limiting assessment of potential sex-related differences during puberty. Additionally, incomplete or inconsistent ICD-10 coding across hospitals may have underestimated the true pathological fraction, with potential effects on refineR model outputs. Individuals aged >85 years were not excluded; however, as shown in the Supplementary Materials, they represented approximately 1% or less of each adult sex-specific parameter group. Therefore, we consider the potential effect of this subgroup on RI estimation to be negligible. Future studies incorporating broader geographic coverage and clinically characterized subgroups (e.g., pregnancy) would further support generalizability and clinical implementation.

## Figures and Tables

**Table 1:** Data sufficiency and proportion of pathological results by test and age group.

Dataset	Demographic Group	Total Number of Records	Number of Thyroid Related Diseases	Fraction of Thyroid Related Diseases
TSH	Total	120,798	4609	3.81%
TSH	Women ≥18 years	67,764	3678	5.42%
TSH	Men ≥18 years	27,111	644	2.37%
TSH	Children 1–14 years	18,524	153	0.82%
TSH	Adolescents 14–18 years	7399	119	1.61%
FT4	Total	120,710	4601	3.81%
FT4	Women ≥18 years	65,941	3678	5.58%
FT4	Men ≥18 years	25,989	636	2.45%
FT4	Girls 1–15 years	11,776	111	0.94%
FT4	Boys 1–15 years	10,757	66	0.61%
FT4	Adolescents 15–18 years	6247	95	1.51%
FT3	Total	37,107	2423	6.54%
FT3	Women ≥18 years	25,638	1948	7.61%
FT3	Men ≥18 years	9493	322	3.40%
FT3	Children 1–6 years	220	27	12.27%
FT3	Children 6–12 years	380	31	8.16%
FT3	Adolescents 12–18 years	1376	95	6.90%

TSH, thyroid-stimulating hormone; FT3, free triiodothyronine; FT4, free thyroxine.

## Data Availability

The data underlying this article will be shared on reasonable request to the corresponding author.

## Supplementary Materials

The following supporting information can be downloaded at https://www.mdpi.com/article/10.3390/diagnostics16121800/s1, Figure S1: Graphical representations of refineR model estimations for TSH and free T4 across the predefined pediatric age groups. Histograms show the observed distributions of test results, and solid curves represent the fitted reference distributions. Vertical dashed lines indicate the estimated lower and upper reference limits together with their 95% confidence intervals.

## Abbreviations

The following abbreviations are used in this manuscript:

RI	Reference interval
CLSI	Clinical and Laboratory Standards Institute
TSH	Thyroid-stimulating hormone
FT3	Free triiodothyronine
FT4	Free thyroxine
USP	United States Pharmacopeia
CV, %	Coefficient of variation
EQA	External quality assessment
ICD	International Classification of Diseases
modBox–Cox	Modified Box–Cox model
IU	International unit

## References

[1] Ozarda Y., Higgins V., Adeli K. (2019). Verification of reference intervals in routine clinical laboratories: Practical challenges and recommendations. Clin. Chem. Lab. Med..

[2] Płaczkowska S., Terpińska M., Piwowar A. (2022). Establishing laboratory-specific RIs for TSH and fT4 by use of the indirect Hoffman method. PLoS ONE.

[3] Clinical and Laboratory Standards Institute (2010). Defining, Establishing, and Verifying Reference Intervals in the Clinical Laboratory.

[4] Jones G.R.D., Haeckel R., Loh T.P., Sikaris K., Streichert T., Katayev A., Barth J.H., Ozarda Y., Null N. (2019). Indirect methods for reference interval determination—Review and recommendations. Clin. Chem. Lab. Med..

[5] Inal T.C., Serteser M., Coşkun A., Ozpinar A., Unsal I. (2010). Indirect RIs estimated from hospitalized population for thyrotropin and free thyroxine. Croat. Med. J..

[6] Haeckel R., Wosniok W., Streichert T. (2021). Review of potentials and limitations of indirect approaches for estimating reference limits/intervals of quantitative procedures in laboratory medicine. J. Lab. Med..

[7] Lo Sasso B., Vidali M., Scazzone C., Agnello L., Ciaccio M. (2019). Reference interval by the indirect approach of serum thyrotropin (TSH) in a Mediterranean adult population and the association with age and gender. Clin. Chem. Lab. Med..

[8] Ammer T., Schützenmeister A., Prokosch H.U., Zierk J., Rank C.M., Rauh M. (2022). RIbench: A proposed benchmark for the standardized evaluation of indirect methods for reference interval estimation. Clin. Chem..

[9] Lorde N., Elgharably A., Kalaria T. (2023). Impact of variation between assays and reference intervals in the diagnosis of endocrine disorders. Diagnostics.

[10] Adeli K., Yu J., Riddell K., Zhang R., Landon O., Jung B. (2025). Comprehensive Age- and Sex-Specific Pediatric Reference Intervals for CLIA Immunoassays on the Snibe MAGLUMI X8 Platform. Clin. Chem. Lab. Med..

[11] Clerico A., Trenti T., Aloe R., Dittadi R., Rizzardi S., Migliardi M., Musa R., Dipalo M., Prontera C., Masotti S. (2019). A multicenter study for the evaluation of the reference interval for TSH in Italy (ELAS TSH Italian Study). Clin. Chem. Lab. Med..

[12] Arzideh F., Wosniok W., Haeckel R. (2011). Indirect reference intervals of plasma and serum thyrotropin (TSH) concentrations from intra-laboratory data bases from several German and Italian medical centres. Clin. Chem. Lab. Med..

[13] Ammer T., Schützenmeister A., Rank C.M., Doyle K. (2023). Estimation of reference intervals from routine data using the refineR algorithm—A practical guide. J. Appl. Lab. Med..

[14] Mirjanic-Azaric B., Avram S., Stojakovic-Jelisavac T., Stcojanovic D., Petkovic M., Bogavac-Stanojevic N., Ignjatovic S., Stojanov M. (2017). Direct estimation of reference intervals for thyroid parameters in the Republic of Srpska. J. Med. Biochem..

[15] Lapić I., Šegulja D., Jakoplić Ž., Lukić I., Rogić D. (2025). Reference intervals and cut-off values for thyroid tests in the Croatian adult population on the Snibe MAGLUMI X6 immunoassay analyzer. Diagnostics.

[16] Xing D., Liu D., Li R., Zhou Q., Xu J. (2021). Factors influencing the reference interval of thyroid-stimulating hormone in healthy adults: A systematic review and meta-analysis. Clin. Endocrinol..

[17] Hollowell J.G., Staehling N.W., Flanders W.D., Hannon W.H., Gunter E.W., Spencer C.A., Braverman L.E. (2002). Serum TSH, T4, and thyroid antibodies in the United States population (1988–1994): NHANES III. J. Clin. Endocrinol. Metab..

[18] Yildiz Z., Dağdelen L.K. (2023). Reference intervals for thyroid disorders calculated by indirect method and comparison with reference change values. Biochem. Med..

[19] Milinković N., Ignjatović S., Žarković M., Radosavljević B., Majkić-Singh N. (2014). Indirect estimation of reference intervals for thyroid parameters. Clin. Lab..

[20] Garmendia Madariaga A., Santos Palacios S., Guillén-Grima F., Galofré J.C. (2014). The incidence and prevalence of thyroid dysfunction in Europe: A meta-analysis. J. Clin. Endocrinol. Metab..

[21] Gunnarsdóttir I., Brantsæter A.L. (2023). Iodine: A scoping review for Nordic Nutrition Recommendations 2023. Food Nutr. Res..

[22] Tanticharoenkarn S., Pipatnavakij P., Piyasuwanying L., Srichomkwan P., Snabboon T., Ganokroj P. (2025). Reference Intervals of Thyrotropin, Thyroid Hormones, and Thyroid Autoantibodies in Adult and Older Individuals According to Iodine Status. EJIFCC.

[23] Wei Y., Zhen J., Hu L., Gu Y., Liu Y., Guo X., Yang Z., Zheng H., Cheng S., Wei F. (2024). Genome-wide association studies of thyroid-related hormones, dysfunction, and autoimmunity among 85,421 Chinese pregnancies. Nat. Commun..

[24] Wang X., Li Y., Zhai X., Wang H., Zhang F., Gao X., Liu S., Teng W., Shan Z. (2021). Reference intervals for serum thyroid-stimulating hormone based on a recent nationwide cross-sectional study and meta-analysis. Front. Endocrinol..

[25] Li Q., Tang Y., Yu X., Qin G., Tian L., Cheng L., Lu Y., Zhao Z., Liu L., Zhang K. (2025). Thyroid function reference intervals by age, sex, and race: A cross-sectional study. Ann. Intern. Med..

[26] Dirks N.F., den Elzen W.P.J., Hillebrand J.J., Jansen H.I., Boekel E.T., Brinkman J., Buijs M.M., Demir A.Y., Dijkstra I.M., Endenburg S.C. (2024). Should we depend on reference intervals from manufacturer package inserts?. Clin. Chem. Lab. Med..

