# Supplementary material for: Age- and Sex-Specific Reference Intervals for TSH, FT4, and FT3 Derived from the Turkish Multi-Center Cohort
Source: Diagnostics (Basel). 2026 Jun 11;16(12):1800. doi: 10.3390/diagnostics16121800 (PMC13298108; doi:10.3390/diagnostics16121800)
Supplement: Supplementary file 1 [file diagnostics-16-01800-s001.zip › supplementary material.pdf]

Reference Interval Estimation

Analyte	QC level	Sincan (%CV)	Mamak (%CV)	Pursaklar (%CV)
TSH	Level 1 (low)	4.33	2.78	3.41
TSH	Level 2 (high)	3.29	2.45	3.63
Free T4	Level 1 (low)	6.88	7.93	4.28
Free T4	Level 2 (high)	3.74	5.22	2.49
Free T3	Level 1 (low)	5.34	5.94	5.23
Free T3	Level 2 (high)	3.69	3.91	4.55

CV, coefficient of variation; QC, quality control; TSH, thyroid-stimulating hormone; FT4, free thyroxine; FT3, free triiodothyronine.

%CV values represent within-laboratory analytical imprecision, calculated from routine internal quality control measurements at two concentration levels. All analyses were performed on Snibe Maglumi analyzers according to the manufacturer’s instructions.

ICD-10 codes list:

- **E00-E07** Disorders of thyroid gland
  - **E00** Congenital iodine-deficiency syndrome
    - **E00.0** Congenital iodine-deficiency syndrome, neurological type
    - **E00.1** Congenital iodine-deficiency syndrome, myxoedematous type
    - **E00.2** Congenital iodine-deficiency syndrome, mixed type
    - **E00.9** Congenital iodine-deficiency syndrome, unspecified
  - **E01** Iodine-deficiency-related thyroid disorders and allied conditions
    - **E01.0** Iodine-deficiency-related diffuse (endemic) goitre
    - **E01.1** Iodine-deficiency-related multinodular (endemic) goitre
    - **E01.2** Iodine-deficiency-related (endemic) goitre, unspecified
    - **E01.8** Other iodine-deficiency-related thyroid disorders and allied conditions
  - **E02** Subclinical iodine-deficiency hypothyroidism
  - **E03** Other hypothyroidism
    - **E03.0** Congenital hypothyroidism with diffuse goitre
    - **E03.1** Congenital hypothyroidism without goitre
    - **E03.2** Hypothyroidism due to medicaments and other exogenous substances
    - **E03.3** Postinfectious hypothyroidism
    - **E03.4** Atrophy of thyroid (acquired)

- **E03.5** Myxoedema coma
- **E03.8** Other specified hypothyroidism
- **E03.9** Hypothyroidism, unspecified

- **E04** Other nontoxic goitre
  - **E04.0** Nontoxic diffuse goitre
  - **E04.1** Nontoxic single thyroid nodule
  - **E04.2** Nontoxic multinodular goitre
  - **E04.8** Other specified nontoxic goitre
  - **E04.9** Nontoxic goitre, unspecified
- **E05** Thyrotoxicosis [hyperthyroidism]
  - **E05.0** Thyrotoxicosis with diffuse goitre
  - **E05.1** Thyrotoxicosis with toxic single thyroid nodule
  - **E05.2** Thyrotoxicosis with toxic multinodular goitre
  - **E05.3** Thyrotoxicosis from ectopic thyroid tissue
  - **E05.4** Thyrotoxicosis factitia
  - **E05.5** Thyroid crisis or storm
  - **E05.8** Other thyrotoxicosis
  - **E05.9** Thyrotoxicosis, unspecified
- **E06** Thyroiditis
  - **E06.0** Acute thyroiditis
  - **E06.1** Subacute thyroiditis
  - **E06.2** Chronic thyroiditis with transient thyrotoxicosis
  - **E06.3** Autoimmune thyroiditis
  - **E06.4** Drug-induced thyroiditis
  - **E06.5** Other chronic thyroiditis
  - **E06.9** Thyroiditis, unspecified

- **E07** Other disorders of thyroid
  - **E07.0** Hypersecretion of calcitonin
  - **E07.1** Dyshormogenetic goitre
  - **E07.8** Other specified disorders of thyroid
  - **E07.9** Disorder of thyroid, unspecified

Distribution of individuals aged >85 years by TSH, fT4, fT3 parameter and sex.

Parameter / group	Total n	n aged >85 years	% aged >85 years
TSH, Women ≥18 years	67,764	681	1.00%
TSH, Men ≥18 years	27,111	256	0.94%
FT4, Women ≥18 years	65,941	708	1.07%
FT4, Men ≥18 years	25,989	260	1.00%
FT3, Women ≥18 years	25,638	185	0.72%
FT3, Men ≥18 years	9,493	72	0.76%

TSH, thyroid-stimulating hormone; FT4, free thyroxine; FT3, free triiodothyronine.
