# Supplementary figures and images for: Age- and Sex-Specific Reference Intervals for TSH, FT4, and FT3 Derived from the Turkish Multi-Center Cohort
Source: Diagnostics (Basel). 2026 Jun 11;16(12):1800. doi: 10.3390/diagnostics16121800 (PMC13298108; doi:10.3390/diagnostics16121800)

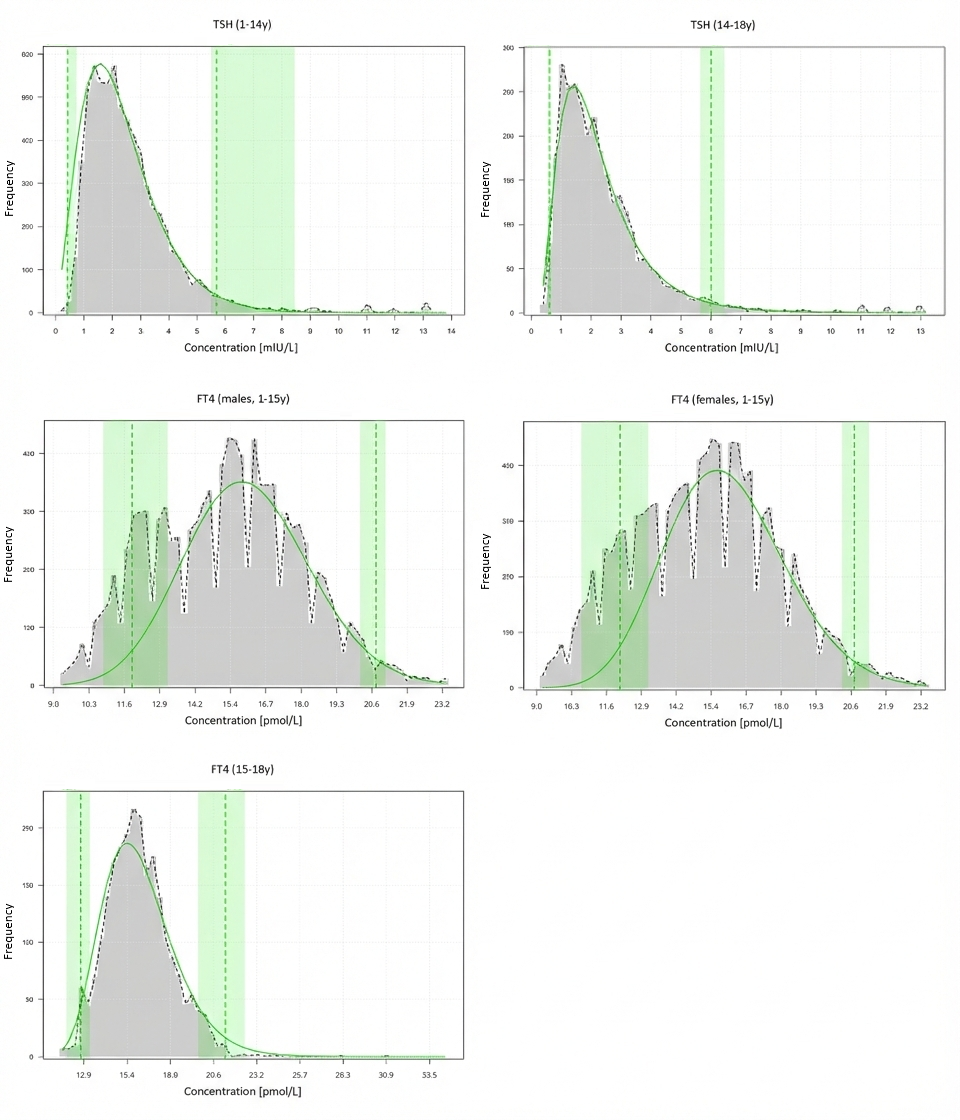

Supplement: Supplementary file 1 [file diagnostics-16-01800-s001.zip › Supplementary Figure S1 .png]
